# Lambert-Eaton Myasthenic Syndrome Secondary to Nivolumab and Ipilimumab in a Patient with Small-Cell Lung Cancer

**DOI:** 10.1155/2019/5353202

**Published:** 2019-07-02

**Authors:** Kavita Agrawal, Nirav Agrawal

**Affiliations:** Department of Internal Medicine, Overlook Medical Center, Summit, NJ 07901, USA

## Abstract

We present a case of a 59-year-old male with a confirmed diagnosis of small-cell lung cancer (SCLC). He had progressive disease even after four cycles of cisplatin and etoposide chemotherapy and 21 cycles of radiation. He was therefore started on immunotherapy with nivolumab every 2 weeks and ipilimumab every 6 weeks. After 4 months of starting immunotherapy, he reported extreme fatigue, muscular weakness, and poor appetite. He was diagnosed with hypothyroidism, primary adrenal insufficiency, and Lambert-Eaton Myasthenic Syndrome (LEMS). LEMS can be both a paraneoplastic syndrome of SCLC and an adverse effect of immunotherapy. Currently, there is no diagnostic test available to determine if a case of LEMS is a paraneoplastic syndrome or immunotherapy-related adverse effect. In our patient, we felt that LEMS was an immunotherapy-related adverse effect rather being a paraneoplastic syndrome. Our determination was based on the time of onset of muscular weakness, presence of other immunotherapy-mediated adverse events, and the appearance of symptoms in spite of SCLC that had been stabilized on immunotherapy. Accordingly, immunotherapy was stopped and a brief tapering course of steroids was initiated. Our patient's muscular weakness from LEMS responded well. His clinical improvement persisted even with radiologic progression of disease after cessation of immunotherapy.

## 1. Introduction

With increasing use of immunotherapy, a wide range of neurological adverse events have been reported. The estimated incidence is reported around 1-2 percent. LEMS secondary to immunotherapy is extremely rare. In our literature review, we could only find one reported case of LEMS secondary to nivolumab. We report here a rare case of immunotherapy-mediated LEMS in a patient with SCLC treated with nivolumab and ipilimumab. It highlights that LEMS should be considered as potential neurological adverse event in patients receiving immunotherapy.

## 2. Case Report

A 59-year-old male presented with complaint of persistent nonproductive cough for 2 months. He had past surgical history of appendectomy. He was a current smoker with smoking history of half to one pack per day since joining high school. His family history included prostate cancer in the father.

Vitals were within normal limits. Physical examination was normal. Chest x-ray posteroanterior and lateral view showed ill-defined mass-like region of airspace opacification within the right hilar and perihilar region. CT scan of the chest without contrast revealed right hilar mass measuring at least 7 to 8 cm in diameter extending into the superior segment of the right lower lobe, encircling the right mainstem bronchus and extending into the subcarinal and precarinal region. Extensive mediastinal, precarinal, and subcarinal lymphadenopathy was also present. These findings raised concern for primary lung cancer. PET/CT skull to thigh with oral and intravenous contrast showed FDG avid mass extending from the right hilum into the right lower lobe measuring approximately 6.8 cm with SUV maximum of 11.6. It also showed FDG avid mediastinal nodes with SUV maximum of 11.9, which was consistent with malignancy. There was no disease reported outside of the thorax. Subsequently, he underwent right-sided VATS and mediastinoscopy with several biopsy samples from mediastinal lymph nodes. The pathology showed sheets of round malignant cells with sparse cytoplasm. The nucleus consisted of fine granular chromatin with indistinct nucleoli. Immunohistochemistry studies showed expression of TTF1 (weak), CAM5.2, CD56, and synaptophysin. Ki67 showed >97% positive staining. The findings were consistent with small-cell lung cancer. Magnetic resonance imaging (MRI) of brain showed no evidence of metastatic disease.

The patient was started on chemotherapy with cisplatin and etoposide with thoracic radiation. He received a total of 4 cycles of chemotherapy and 21 sessions of thoracic radiation. PET/CT was obtained to assess response to the treatment. It showed decrease in size and FDG uptake of primary right lobe mass. However, it showed new hypermetabolic right apical lung lesion and bilateral supraclavicular lymph nodes consistent with disease progression. He was therefore started on systemic immunotherapy consisting of nivolumab 240 mg fixed dose every 2 weeks and ipilimumab 1 mg/kg every 6 weeks.

After 4 months of starting immunotherapy, the patient complained of extreme fatigue and loss of appetite of one-week duration. Thyroid function test (TFT) results are shown in Table 1. The findings were consistent with hypothyroidism. He was started on levothyroxine 75 mcg. On follow-up visit after 1 week of starting levothyroxine, he continued to have fatigue. Also on the clinic visit, he was hypotensive with recorded blood pressure (BP) of 98/68 mmHg. At this point, there was a concern for pituitary or adrenal insufficiency. Laboratory work-up is shown in Table 1. The findings were consistent with primary adrenal insufficiency. He was started on oral hydrocortisone 20 mg in the morning and 10 mg in the evening. After one week of starting hydrocortisone, the patient reported improvement in fatigue and appetite. BP had normalized. Interval CT chest showed stable disease from previous CT scan.

After 8 weeks of starting hydrocortisone, the patient reported new weakness. He complained of difficulty with walking and arm weakness. He could not throw a ball to his dog, which he was able to do previously. He denied double vision, ptosis, dyspnea, chewing, or swallowing difficulties. He denied any bladder or bowel problems. On neurologic examination, CN 2-12 were grossly normal. He was oriented to person, place, and time. Recent and remote memory was normal. Language was fluent with normal comprehension and repetition. Fund of knowledge was normal. On motor exam, weakness of hip flexors bilaterally was noted. Muscle tone, bulk, and strength in other muscle groups were normal. Sensation was normal. Upper and lower extremity reflexes were absent bilaterally. There was facilitation of reflexes with exercise. Finger to nose was normal. Gait was slightly waddling.

Repeat TFT and cortisol level was within normal limits on levothyroxine and hydrocortisone supplementation, respectively. Creatine phosphokinase (CPK) was normal. Acetylcholine receptor binding antibody was normal. Paraneoplastic panel showed elevated P/Q type voltage gated calcium channel antibodies. Brief nerve conduction study showed facilitation of the right medial nerve compound muscle action potential (CMAP) with exercise. MRI brain showed no pathologic intracranial enhancement, mass effect, or recent infarct. A diagnosis of Lambert-Eaton Myasthenic Syndrome was made. Immunotherapy was discontinued. He was started on pyridostigmine 60 mg three times a day. However, intolerable diarrhea ensued and pyridostigmine was discontinued. He was transitioned to prednisone 60 mg daily with taper. The patient noted improvement in leg and arm weakness with prednisone. However, repeat PET scan showed increase in size and activity of left cervical lymphadenopathy and multiple new hypermetabolic hepatic lesions measuring up to 2.5 cm compatible with metastasis. He was initiated on palliation chemotherapy with weekly single agent taxol.

## 3. Discussion

Small-cell lung cancer (SCLC) accounts for 15% of all lung malignancies. It occurs predominantly in smokers. SCLC is considered highly sensitive to chemotherapy and radiation (which is the first-line treatment). Immunotherapy, such as immune checkpoint inhibitors (ICI), can be used as second-line treatments in patients with disease progression on chemotherapy. In the multicenter open phase I/II CheckMate 032 trial, combination therapy with nivolumab and ipilimumab showed better objective response compared to either nivolumab or ipilimumab alone [1]. However, the incidence of adverse events is increased with combination therapy. The underlying mechanism of adverse events is speculated to be immune-mediated.

The most common adverse events include dermatologic toxicity. It can manifest as maculopapular erythematous rash, vitiligo, oral mucositis, or alopecia. Rarely, it can present with severe rash such as Stevens-Johnson syndrome or toxic epidermal necrolysis. Dermatologic toxicity is the earliest adverse event related to ICI treatment [2]. It occurs about 4 weeks after initiation of treatment [2]. Other adverse events include colitis, hepatotoxicity, pneumonitis, and endocrinopathies. Colitis most commonly manifests 6 weeks after the initiation of ICI treatment [3]. Hepatotoxicity commonly presents as elevation in aspartate aminotransferase (AST) and alanine aminotransferase (ALT), rarely with elevation in serum bilirubin. The study has shown that about 20% of patients treated with nivolumab and ipilimumab experienced elevations in AST and ALT more than five times the upper limit of normal [4]. Hepatotoxicity is seen 8 to 12 weeks after the initiation of ICI treatment [4]. Drug-induced pneumonitis is less commonly seen. The study has reported an incidence of 5% in patients treated with monotherapy versus 10% with combination therapy [5]. It can commonly manifest between 2 and 5 months after initiation of ICI treatment [5]. Endocrinopathies include inflammation of thyroid, pituitary, or adrenal glands. They can manifest with nonspecific clinical symptoms such as fatigue, headache, and visual disturbances. Primary adrenal insufficiency and type 1 diabetes mellitus are very rare adverse events. The meta-analysis reported incidence of 0.7% and 0.2%, respectively [6].

With increasing use of ICI, a wide range of neurological adverse events have been reported. The estimated incidence is reported around 1-2 percent [7, 8]. The adverse effects include myopathies, peripheral neuropathy, cerebellar ataxia, autonomic retinopathy, and headache. Other neurologic complications such as myasthenia gravis, posterior reversible encephalopathy syndrome (PRES), and autoimmune encephalitis have also been reported [7, 8].

Our patient developed LEMS after receiving combination therapy of 12 cycles of nivolumab and 4 cycles of ipilimumab. Our case presented a challenge in determining if LEMS was secondary to SCLC or ICI. Determining the etiology was important because if SCLC was the cause of LEMS (and we mistakenly stopped ICI to control LEMS), the patient would have had progression of both SCLC and LEMS. Currently, there is no testing available to help identify if a particular adverse event is ICI-mediated or not. There is ongoing research to find immunologic biomarkers to assess the risk of ICI-mediated adverse events and to aid in the early identification of adverse events [9, 10].

The incidence and prevalence of LEMS as a paraneoplastic neurologic syndrome in patients with SCLC is 3% [11]. In most patients, diagnosis of LEMS precedes the diagnosis of SCLC. Titulaer MJ et al. conducted a study with median follow-up of 8 years in patients with LEMS for the presence of SCLC. The study found that, in 96% of patients, SCLC was found within 12 months of diagnosis of LEMS [11]. Kao JC et al. showed that neurological complications associated with anti-PD 1 inhibitor (nivolumab or pembrolizumab) occur after a median of 5.5 cycles of immunotherapy with a range of 1 to 20 cycles [12]. The cumulative incidence of neurological adverse effects with either nivolumab, ipilimumab, or pembrolizumab when used as single-agent immunotherapy is reported to be less than 1%. Spain L et al. studied incidence of neurological adverse events from ICI in patients with melanoma [13]. The study found that with combination therapy of nivolumab and ipilimumab the incidence of neurological adverse events is increased to 14% [13].

In our literature search, we also found one case of LEMS in a patient with squamous cell lung cancer which was attributed to ICI [14]. In this case, the patient had squamous cell lung cancer which is less commonly associated with LEMS. Also, the onset of neurological complications was more than 2 years after the diagnosis of lung cancer and 20 weeks after the initiation of nivolumab. Radiographically, underlying cancer was in partial remission. These factors made authors conclude that LEMS was ICI-mediated.

In our case, the time of onset of neurological symptoms was 15 months after the diagnosis of SCLC and after 12 cycles of nivolumab and 4 cycles of ipilimumab. Our patient was receiving combination therapy with nivolumab and ipilimumab, which considerably increases the risk of neurological adverse effects [13]. Also, our patient suffered from other ICI-related adverse events such as thyroid dysfunction and primary adrenal insufficiency. All these factors favored ICI as the cause of LEMS. On the other hand, the persistence of stable SCLC disease on radiologic imaging (as opposed to its regression) was a factor that favored the likelihood that LEMS was SCLC-related. However, based on the preponderance of clinical indicators, our assessment was that LEMS in our patient was ICI-mediated. The decision was made to permanently stop immunotherapy. Initially, the patient was started on pyridostigmine for management of LEMS. However, he could not tolerate it due to diarrhea. Then he was started on steroid taper with remarkable improvement noted in the neurological symptoms. However, later in the treatment course, radiographically he had worsening of disease burden. Our patient had improvement in LEMS after stopping immunotherapy and with worsening disease burden. This further supported our assessment that LEMS was ICI-mediated in our patient.

## 4. Conclusion

Our report illustrates an unusual case of LEMS secondary to combination therapy of nivolumab and ipilimumab in a patient with SCLC. It is crucial to identify signs and symptoms of ICI-mediated adverse events. This case highlights that LEMS should be considered as a potential ICI-mediated neurological adverse event.

## Figures and Tables

**Table 1:** Laboratory values.

Test	Result	Reference range
TSH	19.7 *μ*IU/ml	0.340-4.820
Serum T4	1.9 *μ*g/dl	4.7-1.33
Free T4	<0.20 ng/dl	0.59-1.80
Serum T3	45 ng/dl	60-181
Random Cortisol	<0.5 *μ*g/dl	
ACTH	100 pg/ml	7.2-6.3 (6 AM collection)
Prolactin	15.8 ng/ml	2.1-17.7
IGF-I	148 ng/ml	34-232
Testosterone	263 ng/dl	87-780
FSH	9.2 mIU/ml	Males, 13-70 years (1.4-18.1)
LH	3.5 mIU/ml	Males, 20-70 years (1.5-9.3)

## References

[1] Antonia S. J., López-Martin J. A., Bendell J. (2016). Nivolumab alone and nivolumab plus ipilimumab in recurrent small-cell lung cancer (CheckMate 032): a multicentre, open-label, phase 1/2 trial. *The Lancet Oncology*.

[2] Weber J. S., Kähler K. C., Hauschild A. (2012). Management of immune-related adverse events and kinetics of response with ipilimumab. *Journal of Clinical Oncology*.

[3] Weber J. S., Dummer R., de Pril V., Lebbé C., Hodi F. S., MDX010-20 Investigators (2013). Patterns of onset and resolution of immune-related adverse events of special interest with ipilimumab: detailed safety analysis from a phase 3 trial in patients with advanced melanoma. *Cancer*.

[4] Hammers H. J., Plimack E. R., Infante J. R. (2014). Phase I study of nivolumab in combination with ipilimumab in metastatic renal cell carcinoma (mRCC). *Journal of Clinical Oncology*.

[5] Naidoo J., Wang X., Woo K. M. (2017). Pneumonitis in patients treated with anti–programmed death-1/programmed death ligand 1 therapy. *Journal of Clinical Oncology*.

[6] Barroso-Sousa R., Barry W. T., Garrido-Castro A. C. (2018). Incidence of endocrine dysfunction following the use of different immune checkpoint inhibitor regimens. *JAMA Oncology*.

[7] Elrington G. M., Murray N. M., Spiro S. G., Newsom-Davis J. (1991). Neurological paraneoplastic syndromes in patients with small cell lung cancer. A prospective survey of 150 patients.. *Journal of Neurology, Neurosurgery & Psychiatry*.

[8] Wick W., Hertenstein A., Platten M. (2016). Neurological sequelae of cancer immunotherapies and targeted therapies. *The Lancet Oncology*.

[9] Callahan M. K., Yang A., Tandon S. (2011). Evaluation of serum IL-17 levels during ipilimumab therapy: correlation with colitis. *Journal of Clinical Oncology*.

[10] Shahabi V., Berman D., Chasalow S. D., Wang L., Tsuchihashi Z. (2013). Gene expression profiling of whole blood in ipilimumab-treated patients for identification of potential biomarkers of immune-related gastrointestinal adverse events. *Journal of Translational Medicine*.

[11] Titulaer M. J., Wirtz P. W., Willems L. N., van Kralingen K. W., Smitt P. A., Verschuuren J. J. (2008). Screening for small-cell lung cancer: a follow-up study of patients with lambert-eaton myasthenic syndrome. *Journal of Clinical Oncology*.

[12] Kao J. C., Liao B., Markovic S. N. (2017). Neurological complications associated with anti–programmed death 1 (PD-1) antibodies. *JAMA Neurology*.

[13] Spain L., Walls G., Julve M. (2017). Neurotoxicity from immune-checkpoint inhibition in the treatment of melanoma: a single centre experience and review of the literature. *Annals of Oncology*.

[14] Nakatani Y., Tanaka N., Enami T., Minami S., Okazaki T., Komuta K. (2018). Lambert-eaton myasthenic syndrome caused by nivolumab in a patient with squamous cell lung cancer. *Case Reports in Neurology*.

